# Depression after acute traumatic injuries in children

**DOI:** 10.1192/j.eurpsy.2021.646

**Published:** 2021-08-13

**Authors:** Y. Sidneva, S. Valiullina, E. Lvova

**Affiliations:** The Department Of Rehabilitation, Clinical and Research Institute of Emergency Pediatric Surgery and Trauma (CRIEPST), Moscow, Russian Federation

**Keywords:** Depression, Children, traumatic injuries, Rehabilitation

## Abstract

**Introduction:**

Acute traumatic injuries in children are diverse: skeletal trauma, traumatic brain injury (TBI), spinal injury (SCI), amputations, combined trauma and others. Severe injuries lead to severe disability and desadaptation of the child. It is known that children tolerate hard the awareness and acceptance of their new state.

**Objectives:**

To study emotional disorders after traumatic injuries in children at early stages of rehabilitation.

**Methods:**

159 children up to 18 y.o.: 80 (TBI), 60 (SCI), 19 (amputation, skeletal injury, electro-trauma). Methods: psychopathological, psychological; scales, questionnaires.

**Results:**

In children after severe and moderately severe TBI, depression was detected in 43% as a consequence of injury and recovery of mental activity. In children with SCI, depression was detected in 48% as a reaction to stressful situation. In children with amputation, severe skeletal injury, electro-trauma depression was in 60%, both as a consequence of organic recovery of mental activity and as a reaction to psycho-traumatic situation. In the acute period, children had comprehensive interdisciplinary rehabilitation. Neuropsychiatrist recommended neuropharmacotherapy with antidepressants from the group of serotonin reuptake inhibitors (sertraline), GABA preparations. For psychological support, gestalt correction techniques were used.

**Conclusions:**

After acute trauma, depression in children occupies a significant place in clinical picture. Genesis of depressive disorders can be caused both by organic damage to brain structures and by reaction to psychotraumatic situation. In order to improve rehabilitation effectiveness, to make patient’s returning to usual living environment easier as well as to improve the quality of life, interdisciplinary approach is needed since early stages of rehabilitation and after.

